# Adhesions to Mesh after Ventral Hernia Mesh Repair Are Detected by MRI but Are Not a Cause of Long Term Chronic Abdominal Pain

**DOI:** 10.1155/2016/2631598

**Published:** 2015-12-24

**Authors:** Odd Langbach, Stein Harald Holmedal, Ole Jacob Grandal, Ola Røkke

**Affiliations:** ^1^Department of Gastroenterologic Surgery, Akershus University Hospital, P.O. Box 1000, 1478 Lorenskog, Norway; ^2^Department of Radiology, Akershus University Hospital, P.O. Box 1000, 1478 Lorenskog, Norway; ^3^Department of Radiology, Norwegian Radium Hospital, Oslo University Hospital, P.O. Box 4953, Nydalen, 0424 Oslo, Norway; ^4^Faculty of Medicine, University of Oslo, 0316 Oslo, Norway

## Abstract

*Aim*. The aim of the present study was to perform MRI in patients after ventral hernia mesh repair, in order to evaluate MRI's ability to detect intra-abdominal adhesions. *Materials and Methods*. Single-center long term follow-up study of 155 patients operated for ventral hernia with laparoscopic (LVHR) or open mesh repair (OVHR), including analyzing medical records, clinical investigation with patient-reported pain (VAS-scale), and MRI. MRI was performed in 124 patients: 114 patients (74%) after follow-up, and 10 patients referred for late complaints after ventral mesh repair. To verify the MRI-diagnosis of adhesions, laparoscopy was performed after MRI in a cohort of 20 patients. *Results*. MRI detected adhesions between bowel and abdominal wall/mesh in 60% of the patients and mesh shrinkage in 20–50%. Adhesions were demonstrated to all types of meshes after both LVHR and OVHR with a sensitivity of 70%, specificity of 75%, positive predictive value of 78%, and negative predictive value of 67%. Independent predictors for formation of adhesions were mesh area as determined by MRI and Charlson index. The presence of adhesions was not associated with more pain. *Conclusion*. MRI can detect adhesions between bowel and abdominal wall in a fair reliable way. Adhesions are formed both after open and laparoscopic hernia mesh repair and are not associated with chronic pain.

## 1. Introduction

Ventral hernia mesh repair is a common surgical procedure and may be performed by open or laparoscopic technique. Most patients have favorable outcome after surgery, but some patients experience problems such as pain, discomfort, and hernia recurrences [1]. Hernia recurrence may explain some of the complaints and can be diagnosed by clinical investigation with the supplement of ultrasonography or computed tomography (CT). In many cases, however, there is no detectable cause of the patient's symptoms. The problems in these patients are often assumed to come from neuralgias caused by sutures, inflammatory reaction to mesh fixation materials or mesh, or even intra-abdominal adhesions, even if such causes are difficult to verify. The MRI technology is a sensitive method to diagnose abdominal wall pathology, but also adhesions [2] and is increasingly used in the diagnosis of abdominal disease. Although ultrasound is a dynamic tool, its capacity to detect adhesions is limited to the subsurface of the abdominal wall. CT can detect seroma and can also demonstrate typical adhesion-related complications like strangulated obstruction or bowel ischemia. Even with contrast-enhanced CT scan, adhesions cannot be detected directly in most cases but can be assumed due to scar tissue, bowel conglomerations, and luminal changes. Liberal use of dynamic CT-imagines, however, should be selective due to the radiation exposure.

The aim of the present study was to perform MRI in a clinically defined group of patients after LVHR and OVHR, respectively, in order to evaluate to what extent MRI is able to detect the mesh implant and adhesions between the bowel and the mesh or the abdominal wall. We also wanted to find if adhesions could explain chronic pain after ventral hernia mesh repair.

## 2. Materials and Methods

We conducted a single-center follow-up study of 155 patients after LVHR (*n* = 82/53%) or OVHR (*n* = 73/47%) from January 2000 until November 2010. The follow-up included registration of perioperative data from medical records, clinical investigation of the patients, and evaluation of patient-reported pain in relation to different activity levels. Pain was assessed by using a 100 mm VAS scale, 0 meaning no pain and 100 worst imaginable pain.

**Figure 1:**
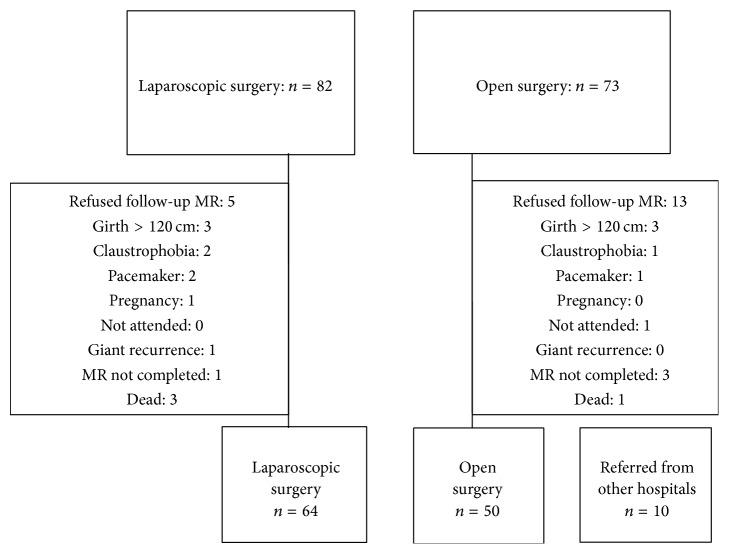
Consort diagram of 124 patients who attended MR-investigation.

Comorbidity was classified according to Charlson et al. [3]. All patients were invited to a magnetic resonance imaging- (MRI-) examination. MRI was finally performed in 114 of these patients (74%), 50 (44%) after OVHR, and 64 (56%) after LVHR, whereas 41 patients were excluded as shown in Figure 1. To increase the number of diagnostic MRI-examinations, another 10 patients, previously undergone ventral hernioplasty with mesh, were included. In these patients data from medical records were not obtained. Thus, MRI was performed in a total of 124 patients.

The MRI study was performed with a 1.5 tesla system (Achieva, Philips Medical Systems, the Netherlands). No premedication or contrast media were administered. First an axial TSE T2-weighted series throughout the abdomen was performed (field of view, 400 mm; matrix, 288 × 200 mm; flip angle, 90°; slice thickness 5 mm) to get an anatomical overview, identify the mesh, and diagnose adhesions between abdominal wall/mesh and bowel. The study was then followed by a cine-MRI, balanced FFE (field of view, 300 mm; matrix, 192 × 224; flip angle, 50°; slice thickness 15 mm). One sequence consisted of 30 dynamic scans in the same position. The patients were asked to increase intra-abdominal pressure and to relax repeatedly throughout this examination. Transverse series covered the abdomen in a craniocaudal direction, sagittal series covering the abdomen from right to left and a few coronal series covered the anterior abdominal wall in an anterior-posterior direction. The distance between every sequence was 15 mm. Depending on the patients size, the number of dynamic scans varied from 400 to 600. The mean examination time was 30 minutes.

**Figure 2:**
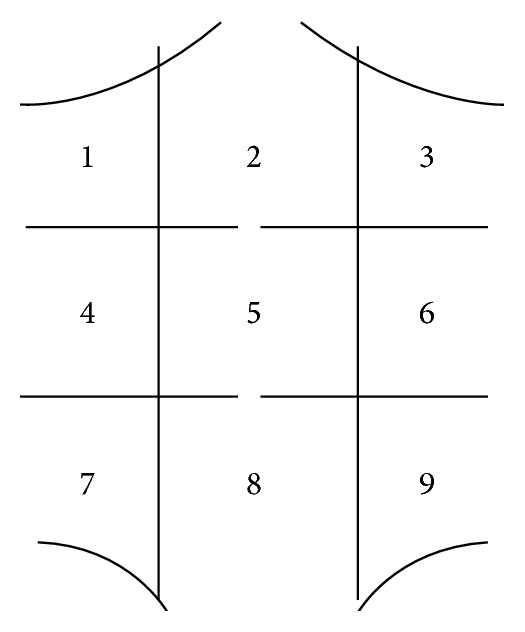
Abdominal map with field segmentation (segments 1–9).

A nine-segment map (Figure 2) was used as localization reference of the abdominal wall. Two experienced radiologists evaluated the MRI-studies in consensus. They were informed that the patients had been operated with mesh hernioplasty for ventral hernia but were blinded to other clinical and per-operative findings. Restricted visceral slide between bowel and adjacent abdominal wall or surgical mesh, with a missing separation between them, had been used as MRI criteria for diagnosis of adhesion. The adhesions were classified according to the location and involved structures. Adhesions between different bowel loops or other organs were not evaluated. Other unrelated abdominal pathology was also recorded.

MRI's true ability of detecting adhesions was validated in a prospective double-blinded study of a cohort of 20 of these patients, in whom laparoscopy was performed after the MRI-scans due to hernia recurrence with complaints.

## 3. Statistics

The analyses were performed on the per-protocol basis. Data in text and tables are given as mean ± standard deviation. Analyses of categorical data were performed by the Pearsons Chi-square test (2-sided) (*n* ≥ 5 in all subgroups) and Fisher's 2-sided exact test (*n* < 5 in any subgroup). The Student's *t*-test was used in analyses of continuous distributed data. Variables associated with the formation of adhesions at the *p* < 0.1 level in the bivariate analyses were included in multivariate analyses using a binary logistic regression model to estimate independent predictors, the odds ratio, and 95% confidence interval. Pearson correlation was used to establish association between variables. Differences between groups are given as actual *p* value and considered different at *p* values below 0.05. The analyses were performed using the SPSS version 22.

## 4. Results 

MRI was performed in 124 patients. Patient characteristics are shown in Table 1. The mesh type used and the technique for mesh repair in 114 patients are shown in Table 2.

The ability of MRI to detect and assess the location of the implanted meshes was dependent on mesh type, with detection rates between 50% and 100%. Information of detectable mesh type and size was available in 68 patients (52%). Size-reduction/shrinkage of mesh occurred in most mesh types and varied between mesh types as shown in Table 3.

106 of the 124 MRI-examinations were evaluable with regard to adhesions between bowel and abdominal wall/mesh. There were variations in patients compliance with respect to deep breath during cine MRI. The breath procedure by the patient was inadequate in 18 patients (15%), in whom evaluation of adhesions could not be performed. Adhesions between bowel and abdominal wall/mesh were described in 63 out of 106 patients (59%) (Table 4) and mostly occurred in the middle and lower midline sectors (sectors 5 and 8). The upper (sectors 1, 2, and 3) and lateral sectors (sectors 4, 6, 7, and 9) had few detectable adhesions as shown in Table 5. 43 patients (41%) were devoid of adhesion on MRI.

In 60 patients, there was available information about the size of the implanted mesh, and MRI could define both the mesh size and the presence or absence of adhesions. In these patients there was a significant correlation between mesh shrinkage and adhesion formation (*p* = 0.003, *R* = 0.374).

The placement of mesh in open surgery was onlay (*n* = 8/16%), sublay (13/26%), and open IPOM (*n* = 29/58%). Adhesions between bowel and abdominal wall/mesh were detected in 59 of 97 patients (60.8%) with evaluable MRI-scans and clinical data. Adhesions were demonstrated regardless of mesh type, however with a variation between 33% and 75% (Table 6). There was no significant difference between laparoscopic and open mesh repair with regard to formation of adhesions. Adhesions were identified in 67% of the patients after LVHR and in 49% after OVHR (Table 7). Adhesions to mesh were detected in 14/29 (48%) of the patients with “open IPOM.”

The diagnosis of adhesions by MRI was validated by laparoscopy in 20 patients (Table 8). Laparoscopy was considered the “gold standard.” 18 patients in this group had evaluable MRI-investigations. In the cohort where the results of MRI were investigated by subsequent laparoscopy, adhesions between bowel and abdominal wall were diagnosed by MRI in nine patients. At laparoscopy, 10 patients had adhesions between bowel and abdominal wall/mesh. MRI diagnosed adhesions between bowel and abdominal wall/mesh in two patients that did not have adhesions at laparoscopy. MRI failed to diagnose adhesions in three patients with adhesions at laparoscopy (Table 8). From this limited cohort, sensitivity of MRI was calculated to 7/10 = 70%, specificity 6/8 = 75%, and positive predictive value. 7/9 = 78%, negative predictive value: 6/9 = 67%. MRI was unable to detect adhesion between the omentum and the abdominal wall. At laparoscopy, 17 patients had such adhesions, mostly in region 5. Furthermore, some kind of adhesions was detected in all patients in one or more regions. Thus, the MRI underestimated the presence of adhesions.

To identify predictors for the genesis of adhesions, factors considered as important were investigated. In univariate analysis, the Charlson index, hernia width, mesh area, and operative time was associated with the presence of adhesions as determined by MRI (Table 9). When tested in a multivariate model, mesh area as determined by MRI and Charlson index were independent predictors of adhesions (Table 10).

The patient-reported pain during average-, normal-, moderate-, and maximal activity at follow-up was determined by the VAS-scale. In the laparoscopic hernia repair group we used nonabsorbable tackers (*n* = 37/58%), nonabsorbable sutures (*n* = 14/22%), or both (*n* = 13/20%). We could not find any significant correlation between type of mesh fixation and chronic pain. There were similar results in patients with adhesions compared to patients without adhesions, except during normal activity, where patients with adhesions reported less pain than patient with adhesions (Table 11). Of the 114 patients, eight (7%) patients reported chronic pain (VAS > 30) during normal activity, six (5%) during average activity, 15 (13%) during moderate activity, 30 (36%) during maximal activity, and seven (6%) reported pain at follow-up. The number of patients with chronic pain during normal activity was lower in the group with adhesions (2%) compared to the group without adhesions (15%) (Table 12).

## 5. Discussion

In the present study, MRI could identify mesh in 77% of patients, with a rage of 50–100% depending on mesh type. Polypropylene allows for tissue ingrowth to an extent that makes detection difficult. Many of the recent meshes, however, like polytetrafluoroethylene mesh (ePTFE), are well detected on MRI. In 28 patients, laparoscopically inserted ePTFE were all visible, whereas inserted polypropylene meshes were not detectable [4]. Laparoscopically placed mesh may be more easily detected than mesh placed by open surgery [5]. In the present study, 57% of the polypropylene meshes were detected, possibly due to a more sensitive MRI-machine.

By MRI, we could demonstrate a size-reduction of 20–50% in mesh-implants depending on mesh types. Parietex composix showed about 50% shrinkage. This is in accordance with previous studies, reporting 20–40% shrinkage [6, 7]. In an experimental study in goats, Zinther et al. could demonstrate a 40% shrinkage of Parietex and 20% shrinkage of DynaMesh three months after insertion, with no further shrinkage thereafter [8]. In a study of polypropylene mesh with radioopaque markers, CT, after mesh insertion and two years postoperatively, demonstrated no shrinkage in 46 out of 50 patients and 3–22% shrinkage in the rest [9]. The observed size-reduction in our study might therefore have other causes than true shrinkage, like bulging and doubling, as previously described [10].

Intra-abdominal adhesions may have deleterious effects, like intestinal obstruction followed by chronic pain and reduced quality of life [1]. Previous studies using MRI after hernia mesh repair have reported adhesion rates of 70−100% [4, 11–13]. Adhesion seems to be associated with abdominal pain and discomfort [11]. The question however remains if these adhesions, in the absence of bowel obstruction, are capable of eliciting pain. In the present study, the rates of MRI diagnosed adhesions between bowel loops and abdominal wall/mesh are in agreement with others [12]. Adhesions to mesh were demonstrated in all mesh types, and there were no differences between patients operated with laparoscopic or open mesh repair. Larger mesh size at MRI was associated with higher degree of adhesions. Adhesions are thought to be caused by inflammation. In the present study, many factors were recorded and tested that might contribute to the formation of adhesions. We could demonstrate a significant correlation between mesh shrinkage and presence of adhesions. The implanted mesh may induce an inflammatory reaction, inducing shrinkage and creating adhesions, which in turn may amplify mesh shrinkage. The area of mesh as determined by MRI and Charlson comorbidity index was independent predictors of adhesion formation. Notably, laparoscopic and open mesh repair had similar rates of adhesion formation.

The MRI-investigation was designed to detect adhesions between bowel and abdominal wall/mesh and not between bowel segments. In 15% of patients, the MRI-investigation was not evaluable. During the MRI-scan period for 20–25 minutes, the patient must continuously use the abdominal muscles, which weakens in several patients during the procedure, followed by reduction in intestinal motility, and difficulty in interpretations. In some previous reports, a MRI-slice-thickness of 5–15 mm has been used [1]. To increase patient compliance, the MRI-slice-thickness of 15 mm was selected in the present study to reduce the scan-time, which in theory could overestimate the presence of adhesions.

The presence of adhesions was also associated with experience of pain during normal activity. Surprisingly, patients with adhesions experienced less pain than patients without adhesions. This is in contradiction to the general hypothesis today that adhesions may cause pain. Patients with abdominal pain thought of as being caused by adhesions are often scheduled for surgical adhesiolysis. Some support for this was found in a study by Demco, where laparoscopy was performed in 20 sedated but awake patients, and a systematic traction of adhesions was performed, which induced pain depending on type of adhesions [14]. In the present study, MRI was able to detect adhesions between bowel and abdominal wall. About 6% developed chronic pain at long term follow-up. Patients with adhesions did not have more pain than patients without adhesions. Thus, the present study does not support that adhesions produce pain. This is in accordance with a study by Swank et al., who randomized patients with chronic abdominal pain and adhesions to either laparoscopy with adhesiolysis or laparoscopy alone. There was no difference between the groups, except for more complications after adhesiolysis [15]. Adhesions between omentum and abdominal wall could also be of importance in pain generation, but these adhesions could not be detected with MRI. The study was also not designed to detect adhesions between bowel loops, or between female internal genitals and bowel loops, which also may generate pain. Previous studies have validated the use of MRI. In one study, with intraoperative validation of the MRI's ability to detect adhesions, a prevalence of 96%, an accuracy of 90%, a sensitivity of 93%, a positive predictive value of 96%, and a specificity of 25% were found [16]. The low specificity was explained by the very low number of patients found without adhesions both with cine-MRI and intraoperatively. In a study of preoperative MRI before planned laparotomy, MRI could detect adhesions with a sensitivity of 21.5%, with a specificity of 87% [12]. In the present study the sensitivity was better, and specificity about the same. Interestingly, the* absence* of adhesions may be more accurately defined by ultrasound than by MRI [12], but the* presence* of adhesions is best detected with MRI compared to high-resolution ultrasonography. In a study, intra-abdominal adhesions were determined in 53 patients with MRI and 3 with ultrasonography, where most adhesions were between small bowel and abdominal wall, thereafter bowel-bowel adherences [16]. Only adhesions between intestines and abdominal wall could be detected in the present study. The use of 15 mm slices versus 10 mm slices may be an explanation.

## 6. Conclusion

MRI is a sensitive tool to detect various types of implanted mesh, as well as adhesions between bowel and abdominal wall/mesh with a fair sensitivity and specificity. There is no difference between the tendency to form adhesions after open or laparoscopic mesh repair. The area covered by the mesh is associated with formation of adhesions. Adhesions between bowel and abdominal wall cannot explain chronic pain after laparoscopic or open hernia mesh repair.

## Figures and Tables

**Table 1:** Characteristics of 114 patients investigated with MRI.

	Laparoscopic (*n* = 64)	Open (*n* = 50)	*p*
Age at hernia repair (y)	55.2 ± 14.1	55.1 ± 11.7	0.959
Gender (male : female)	24 : 40	25 : 25	0.181
Charlson score	0.3 ± 0.7	0.4 ± 0.8	0.613
Charlson index	1.6 ± 1.4	1.6 ± 1.4	0.914
BMI at hernia repair (kg/m^2^), mean ± SD	30.1 ± 5.5	29.0 ± 5.0	0.289
BMI at follow-up (kg/m^2^), mean ± SD	29.7 ± 6.1	28.5 ± 5.8	0.241
Area of hernia (cm²)	55 ± 59	40 ± 45	0.123
Area implanted mesh (cm²)	227 ± 115	180 ± 129	0.101
Area mesh determined MR (cm²)	131 ± 79	106 ± 73	0.145
Days in hospital	2.4 ± 1.6	2.4 ± 2.2	0.932
Time from surgery to follow-up (y)	3.8 ± 1.4	4.6 ± 2.3	0.035
Time from follow-up to MRI (y)	0.9 ± 1.3	1.3 ± 2.5	0.222

**Table 2:** Detection of mesh by MRI in 114 patients treated with mesh hernioplasty.

	Laparoscopic		Open		All	
	(*n* = 64)		(*n* = 50)		(*n* = 114)	
	*n*	%	*n*	%	*n*	%
Parietex composix	29/32	91	6/6	100	35/38	92
Polypropylene	1/1	100	11/20	55	12/21	57
Bard Comp.	12/14	86	2/4	50	14/18	78
Goretex dual mesh	7/8	88	6/9	67	13/17	72
Proceed	7/7	100	0	—	7/7	100
Marlex	0	—	3/6	50	3/6	50
TiMESH	2/2	100	1/1	100	3/3	100
Unknown	0	—	1/4	25	1/4	25
SUM	58/64	91	30/50	60	88/114	77

**Table 3:** Size of implanted mesh compared to mesh size at follow-up determined by MRI in patients with clinical data (*n* = 68).

	Area of implanted mesh	Area by MRI	% shrinkage	*p*
All (*n* = 68)	223 cm^2^ ± 115 cm^2^	133 cm^2^ ± 79 cm^2^	−30%	0.000
Parietex composix (*n* = 34)	227 cm^2^ ± 128 cm^2^	117 cm^2^ ± 72 cm^2^	−49%	0.000
Polypropylene (*n* = 4)	99 cm^2^ ± 52 cm^2^	101 cm^2^ ± 96 cm^2^	+1%	0.941
Bard composix (*n* = 13)	240 cm^2^ ± 96 cm^2^	153 cm^2^ ± 74 cm^2^	−36%	0.003
Goretex dual mesh (*n* = 9)	263 cm^2^ ± 123 cm^2^	202 cm^2^ ± 95 cm^2^	−23%	0.110
Proceed (*n* = 4)	217 cm^2^ ± 81 cm^2^	136 cm^2^ ± 56 cm^2^	−37%	0.082
Marlex (*n* = 1)	236 cm^2^	65 cm^2^	−72%	—
TiMESH (*n* = 3)	157 cm^2^ ± 34 cm^2^	75 cm^2^ ± 20 cm^2^	−52%	0.040

**Table 4:** Adhesions between bowel and abdominal wall and/or mesh as determined by MRI in 106 evaluable MRI-investigations.

Adhesions	*n*	%
Small bowel and mesh	32	30
Small bowel and mesh and abdominal wall	4	4
Colon and mesh	4	4
Small bowel and colon and mesh	8	8
Small bowel and colon and mesh and abdominal wall	2	2
Small bowel and abdominal wall	11	10
Small bowel and colon and abdominal wall	2	2
No adhesions	43	40
All	106	100
Not evaluable	18	

**Table 5:** Adhesions between bowel and abdominal wall and/or mesh as determined by MRI in 106 evaluable MRI-investigations.

Region	No adhesions		Adhesions between bowel and abdominal wall/mesh	
	*n*	%	*n*	%
1	103	98%	2	2%
2	96	91%	9	9%
3	103	97%	3	3%
4	104	98%	2	2%
5	59	55%	46	45%
6	103	97%	3	3%
7	99	93%	7	7%
8	71	67%	35	33%
9	105	99%	1	1%

**Table 6:** Adhesions between bowel and mesh as determined by MRI in 97 patients with medical records from the operation and evaluable MR-investigations.

	Adhesions to mesh		Adhesions to abdominal wall		No adhesions		Evaluable		Not evaluable	
	*n*	%	*n*	%	*n*	%	*n*	%	*n*	%
Goretex	6	46	1	8	6	46	13	100	4	23
Parietex composix	20	61	3	9	10	30	33	100	5	13
Titan	1	33	0	0	2	67	3	100	0	100
Proceed	2	29	2	29	3	42	7	100	0	100
Bard composix	9	60	1	7	5	33	15	100	3	17
Polypropylene	6	38	2	12	8	50	16	100	5	25
Marlex	1	17	1	17	4	66	6	100	0	100
Unknown	1	25	2	50	1	25	4	100	0	100
SUM	46	47	12	12	39	40	97	100	17	15

**Table 7:** Adhesions between bowel and mesh/abdominal wall as determined by MRI in 114 patients with medical records after laparoscopic or open mesh repair.

	Laparoscopic		Open		All		*p*
	*n*	%	*n*	%	*n*	%	
Adhesions to mesh	32	55	14	36	46	47	0.063
Adhesions to abdominal wall	7	12	5	13	12	13	
No adhesions	19	33	20	51	39	40	
All evaluable	58	100	39	100	97	100	
Not evaluable	6	9	11	22	17	15	
All	64		50		114		

**Table 8:** Comparison between laparoscopy (considered as gold standard) and MRI.

MRI	Laparoscopy		Sum
	Adhesions	No adhesions	
Adhesions (positive)	7 (true positive)	2 (false positive)	9
No adhesions (negative)	3 (false negative)	6 (true negative)	9
SUM	10	8	18

**Table 9:** Univariate analysis of clinical parameters of possible importance for creations of adhesions in 97 patients with clinical data and evaluable MR-scans.

	Adhesions	No adhesions	*p*
	*n* = 58	*n* = 39	
Age at surgery (years)	57.8 ± 12.1	50.4 ± 13.9	0.007
Charlson score	0.41 ± 0.75	0.21 ± 0.62	0.153
Charlson index	1.8 ± 1.4	1.1 ± 1.4	0.012
BMI (kg/m^2^), mean ± SD	29.0 ± 4.8	29.1 ± 5.3	0.978
Hernia length (preoperative measure) (cm)	6.5 ± 3.7	5.3 ± 3.5	0.121
Hernia width (preoperative measure) (cm)	6.2 ± 3.4	4.7 ± 2.3	0.018
Mesh area (cm^2^)	233.6 ± 130.6	173.6 ± 68.8	0.036
Number of tacker rows	2.25 ± 0.7	2.47 ± 0.7	0.133
Operative time (min)	113.4 ± 56.1	88.2 ± 41.3	0.021
Postoperative stay (d)	2.7 ± 2.3	2.0 ± 1.2	0.064
Time from surgery to follow-up (y)	4.37 ± 1.77	3.92 ± 2.14	0.258
Time from surgery to MRI (y)	5.1 ± 1.8	5.0 ± 2.7	0.971
Area of mesh determined by MRI (cm²)	134.7 ± 79.9	92.6 ± 55.8	0.023
Gender (male/female)	30/28	15/24	0.199
LVHR/OVHR	39/19	19/20	0.141
Postoperative complications (no/yes)	40/18	27/12	0.420

**Table 10:** Multivariate analysis of clinical parameters of possible independent importance for creation of adhesions in 97 patients with clinical data and evaluable MR-scans.

	*B*	S.E.	Wald	*p*	Exp(*B*)
Age at hernia mesh repair	0.039	0.065	0.361	0.548	1.040
Charlson index	−1.813	0.898	4.073	0.044	0.163
Hernia width	0.088	0.195	0.205	0.651	1.092
Mesh area determined at mesh repair	−0.002	0.007	0.113	0.736	0.998
Mesh area determined by MRI	−0.019	0.010	3.851	0.050	0.981
Operative time	−0.006	0.011	0.298	0.585	0.994
Postoperative stay	0.078	0.473	0.027	0.869	1.081

**Table 11:** VAS-scores in patients with and without adhesions between bowel and abdominal wall as determined by MRI in 97 evaluable patients with registered VAS-scores.

	Adhesions	No adhesions	*p*
	(*n* = 58)	(*n* = 39)	
Average pain	2.4 ± 7.0	4.5 ± 13.2	0.317
Pain during normal activity	3.8 ± 8.3	9.9 ± 17.6	0.025
Pain during moderate activity	7.7 ± 12.2	14.0 ± 21.8	0.072
Maximal pain in last 30 days	17.6 ± 20.5	20.9 ± 22.8	0.488
Pain at follow-up (today)	3.9 ± 8.5	6.5 ± 12.6	0.217

**Table 12:** Number of patients with chronic pain (VAS ≥ 30) in 97 evaluable patients with or without adhesions.

	Adhesions	No adhesions	*p*
	(*n* = 58)	(*n* = 39)	
Average pain	1 (2%)	4 (10%)	0.154
Pain during normal activity	1 (2%)	6 (15%)	0.016
Pain during moderate activity	5 (9%)	9 (23%)	0.075
Maximal pain in last 30 days	16 (28%)	12 (31%)	0.820
Pain at follow-up (today)	2 (3%)	4 (10%)	0.216
